# Extended-Spectrum β-Lactamases and/or Carbapenemases-Producing Enterobacteriaceae Isolated from Retail Chicken Meat in Zagazig, Egypt

**DOI:** 10.1371/journal.pone.0136052

**Published:** 2015-08-18

**Authors:** H. M. Abdallah, E. A. Reuland, B. B. Wintermans, N. al Naiemi, A. Koek, A. M. Abdelwahab, A. M. Ammar, A. A. Mohamed, C. M. J. E. Vandenbroucke-Grauls

**Affiliations:** 1 Medical Microbiology and Infection Control, VU University Medical Center, Amsterdam, the Netherlands; 2 Laboratory for Medical Microbiology and Public Health, Hengelo, the Netherlands; 3 Microbiology and Infection Control, Ziekenhuisgroep Twente, Almelo, Amsterdam, the Netherlands; 4 Department of Bacteriology, Mycology and Immunology, Faculty of Veterinary Medicine, Zagazig University, Egypt; Ross University School of Veterinary Medicine, SAINT KITTS AND NEVIS

## Abstract

**Objectives:**

The aim of the present study was to determine the prevalence and to characterize extended-spectrum β-lactamases- and/or carbapenemases-producing Enterobacteriaceae among Enterobacteriaceae isolated from retail chicken meat in Zagazig, Egypt.

**Methods:**

One hundred and six Enterobacteriaceae isolates were collected from retail chicken meat samples purchased in Zagazig, Egypt in 2013. Species identification was done by MALDI-TOF MS. Screening for ESBL-E was performed by inoculation of isolates recovered from meat samples onto the EbSA (Cepheid Benelux, Apeldoorn, the Netherlands) selective screening agar. ESBL production was confirmed by combination disc diffusion test with clavulanic acid (Rosco, Taastrup, Denmark). Carbapenemases production was confirmed with double disk synergy tests. Resistance genes were characterized by PCR with specific primers for TEM, SHV, and CTX-M and carbapenemases (KPC, NDM, OXA-48, IMP and VIM). PCR products of CTX-M genes were purified and sequenced. Phylogenetic grouping of *E*. *coli* was performed by a PCR-based method.

**Results:**

Of these 106 isolates 69 (65.09%) were ESBL producers. Twelve (11.32%) of these isolates were also phenotypically class B carbapenemases producer. TEM genes were detected in 61 (57.55%) isolates. 49 (46.23%) isolates harbored CTX-M genes, and 25 (23.58%) carried genes of the SHV family. All CPE belonged to the NDM group. The predominant CTX-M sequence type was CTX-M-15 (89.80%). The majority (80%) of the ESBL-EC belonged to low virulence phylogroups A and B1.

**Conclusions:**

This is the first study from Egypt reporting high rates of ESBLs and carbapenemases (65.09% and 11.32%, respectively) in Enterobacteriaceae isolated from retail chicken meat. These results raise serious concerns about public health and food safety as retail meat could serve as a reservoir for these resistant bacteria which could be transferred to humans through the food chain.

## Introduction

The β-lactam antibiotics have been amongst the most successful drugs for the treatment of bacterial infections for the past 60 years [1]. They are arguably the most important and widely used antimicrobial class for treating bacterial infections in both human and veterinary medicine, because of their excellent safety profile, broad antimicrobial spectrum, availability of orally bioavailable formulations, and the low cost of many products [2]. More than half of all currently used antibiotics belong to the β-lactam group, but their clinical effectiveness is severely limited by the emergence of β-lactam resistant bacteria [3]. The resistance to β-lactam antibiotics occurs as a result of drug inactivation by β-lactamases, target site (penicillin-binding proteins) alterations, diminished permeability and efflux [4]. In Gram negative pathogens, β-lactamases are the major determinant of this resistance [5]. Extended-spectrum β-lactamases (ESBLs) are a rapidly evolving group of β-lactamases which hydrolyze third-generation cephalosporins and aztreonam but not carbapenems [6]. Extended-spectrum β-lactamase producing Enterobacteriaceae (ESBL-E) are prevalent worldwide [7]. Chicken meat has been proposed to constitute a source for ESBL-E that colonize and infect humans [8]. Close genetic similarities among extended-spectrum β-lactamase-producing *Escherichia coli* (ESBL-EC) isolated from chicken meat and humans together with the concurrent presence of CTX-M-1 and TEM-52 genes on similar plasmids of *Escherichia coli* isolated from both sources support the occurrence of food-borne transmission of ESBL genes [9,10]. Furthermore, ESBL-EC isolated from chicken meat was documented as a source of ESBL-EC in humans [11]. Previous studies reported high ESBL contamination rates of chicken meat in the Netherlands [12,13], Sweden [14] and recently in Germany [8,15]. A recent study demonstrated the presence of carbapenemase-producing Enterobacteriaceae (CPE) in Broiler Chicken Fattening Farms [16] but there are no reports on acquired carbapenemase producers from retail chicken meat [17].

In Egypt, ESBL and/or CPE have been reported in hospitalized patients[18,19]. It is not known, however, whether Egyptian chicken meat is contaminated with ESBL-E and /or CPE. Therefore, we carried out this study to determine the prevalence and to characterize ESBL-E and /or CPE isolated from retail chicken meat in Zagazig, Egypt.

## Materials and Methods

### Bacterial isolates

Over a period of eight weeks between January and March, 2013, seven butcher shops, located in different districts of Zagazig City, Egypt (latitude 30°35′15″ N; longitude 31°30′07″ E and altitude 16 metre above sea level), were visited once a week. At each visit, two random fresh chicken carcasses were bought at each shop, and immediately transported to the laboratory for culture.Sampling was done by whole carcass rinse method [20]. The rinse fluid was collected, plated in parallel on selective EbSA-ESBL Screening Agar [21] for the isolation of bacteria resistant to broad-spectrum cephalosporins and on MacConkey agar for the characterization of the dominant flora. The two plates were incubated aerobically at 37°C for 24 h. A pure colony was picked up from both plates for further identification by Vitek MS system (BioMérieux, Marcy l’Étoile, France).

### Phenotypic screening and confirmation of ESBL-E and CPE

ESBL and carbapenemases production were screened by disk diffusion method on Mueller-Hinton agar using ceftazidime (30 μg), cefotaxime (30 μg), meropenem(10 μg), imipenem (10 μg) and ertapenem (10 μg), and interpreted according to the clinical breakpoints recommended by CLSI and NVMM [22,23]. Confirmation of ESBL production was carried out by the combination disc diffusion test with clavulanic acid (Rosco, Taastrup, Denmark). The inhibition zone around the cephalosporin (cefotaxime, ceftazidime and cefepime) discs combined with clavulanic acid (CA) is compared to the zone around the discs with the cephalosporin alone. A positive test is defined as ≥5 mm increase in zone diameter around the cephalosporin disc with CA in comparison to a disc without [22,23].

Carbapenemases production was confirmed by carbapenemase double disk synergy test [24]. Enhancement of the inhibition zone in the area between the carbapenems (meropenem and /or Imipenem) and the inhibitor-containing disk (3-aminophenylboronic acid (APBA), or dipicolinic acid (DPA)) was considered to be a positive result [25].

### Real-time PCR for characterization of β-lactamase-encoding genes

DNAs of all phenotypic ESBL- and carbapenemases positive isolates were extracted by boiling lysis method as described previously [23,26]. The phenotypic ESBL-positive isolates were analyzed for the presence of genes encoding TEM, SHV and CTX-M by real-time PCR using primers described before [27–29]. Carbapenemases positive isolates were screened for KPC, NDM, OXA-48, IMP and VIM by multiplex PCRs using primers described before [30]. All real-time PCR amplifications and melting curve analysis were carried out on the LightCycler 480 II system with software version 3.5 (Roche, Mannheim, Germany) in a total volume of 20 μl. Amplification conditions were as described elsewhere [31,32].

### DNA sequencing analysis

Purified PCR products of ESBL-E were sequenced with Sanger ABI 3730 XL automated DNA sequencer (BaseClear, Leiden, The Netherlands).The nucleotide sequences were analyzed using the CodonCode Aligner software (Version 5.0.2), compared, and aligned with reference sequences available at the National Center for Biotechnology Information website (www.ncbi.nlm.nih.gov).

### Phylogenetic grouping of *E*.*coli*

*E*. *coli* isolates were allotted to one of the four main phylogenetic groups (A, B1, B2, or D) using a PCR-based method targeted to the *chuA* and *yjaA* genes and the *TspE4*.*C2* DNA fragment, developed by Clermont et al.[33].

## Results

Of the total 112 carcasses collected, cultures of 12 carcasses had to be discarded because of the growth of *Pseudomonas* spp. (n = 7) or Gram positive cocci (n = 5). ESBL-E was found in 63% (63/100) of the carcasses contaminated with Enterobacteriaceae, whereas 12 carcasses harbored CPE. Some carcasses showed growth of more than one species of Enterobacteriaceae, resulting in 106 isolates available for analysis, 69 (65.1%) isolates were ESBL producers (Table 1). The distribution of ESBL-producing species among different Enterobacteriaceae was: 44(63.77%) *Klebsiella pneumoniae*, 10 (14.49%) *E*.*coli*, 13 (18.84%) *Enterobacter cloacae*, and 2 (2.90%) *Klebsiella oxytoca*. Twelve (11.32%) of these isolates were also phenotypically class B carbapenemases-producer. CPE were *Klebsiella pneumoniae* (n = 11), and *Klebsiella oxytoca* (n = 1).

**Table 1:** Prevalence of the different types of ß-lactamase-encoding genes among different *Enterobacteriaceae*.

species	No. of isolates	No. of ESBL positive	TEM alone	TEM + CTX-M	TEM + SHV	TEM + CTX-M + SHV	CTX-M alone	CTX-M + SHV
*Klebsiella pneumoniae*	44	44	11	7	3	20	1	2
*E*.*coli*	38	10	2	4	0	0	4	0
*Enterobacter spp*	21	13	4	9	0	0	0	0
*Klebsiella oxytoca*	2	2	0	1	0	0	1	0
*Citrobacter* spp	1	0	0	0	3	0	0	0
Total	106	69	17	21	3	20	6	2

The TEM gene was detected in 61 (57.55%) isolates. 49 (46.23%) isolates contained CTX-M genes; of these, 47 (95.92%) belonged to CTX-M-1 group (44 CTX-M-15 and 3 unidentified) and 2 (4.08%) belonged to CTX-M-9 group (all were CTX-M-14) and 25 (23.58%) belonged to the SHV family.

20 isolates coproduced TEM, SHV, and CTX-M genes, 21 harboured CTX-M and TEM genes, 2 contained CTX-M and SHV genes, 3 expressed TEM and SHV genes, 6 possessed CTX-M genes alone and 17 produced TEM genes only (see also Table 1). All CPE belonged to the NDM group. The numbers of carcasses contaminated with ESBL-E and/or CPE per each shop during the different sampling period is shown in Table 2.

**Table 2:** Numbers of carcasses contaminated with ESBL-E and/or CPE per each shop during the different sampling period.

Sampling Periods	Shop 1		Shop 2		Shop 3		Shop 4		Shop 5		Shop 6		Shop 7		Total	
	ESBL-E	CPE	ESBL-E	CPE	ESBL-E	CPE	ESBL-E	CPE	ESBL-E	CPE	ESBL-E	CPE	ESBL-E	CPE	ESBL-E	CPE
1	1	0	2	0	1	0	1	0	1	1	2	0	1	1	9	2
2	2	0	1	1	0	0	1	0	1	0	1	0	2	2	8	3
3	1	0	1	0	1	0	0	0	1	0	1	0	1	0	6	0
4	1	1	1	0	2	1	1	0	2	1	0	0	1	0	8	3
5	1	0	2	0	1	0	2	1	1	0	0	0	1	0	8	1
6	1	0	1	0	2	1	1	0	1	0	1	1	1	0	8	2
7	2	0	0	0	0	0	2	0	1	0	1	0	2	1	8	1
8	1	0	1	0	1	0	1	0	2	0	0	0	2	0	8	0
Total	10	1	9	1	8	2	9	1	10	2	6	1	11	4	63	12

Phylogenetic grouping revealed, of the 10 ESBL-EC isolates, 4 belonged to group A, 4 to group B1, 2 to group D and none to group B2. In the ESBL- negative *E*.*coli*, 6 of the isolates belonged to group A, 11 to group B1, 7 to group B2 and 4 to group D.

## Discussion

Our data showed that two thirds of Enterobacteriaceae isolates recovered from chicken meat samples were ESBL positive; more than one in ten isolates were also resistant to carbapenems. To the best of our knowledge, this is the first study conducted to determine the prevalence and to characterize ESBL-E and /or CPE isolated from retail chicken meat in Egypt. Nearly similar results were found in Spain, where 67% of the chicken meat was reported to be contaminated with ESBL or ESBL-like resistance genes [34]. However, higher rates of ESBL-E in chicken meat were reported in Switzerland [35] and The Netherlands [12,13]. On the other hand, lower rates of ESBL-E were found in chicken meat in Gabon [36] and Germany [37].

The detection of high numbers of carbapenem-resistant isolate isolates harboring NDM raises serious concerns about public health since carbapenems are considered the first-line drugs for the treatment of serious infections due to ESBL-producing bacteria[39]. Carbapenemase-producing isolate isolates have been detected in poultry farms but there are no reports on acquired carbapenemase producers from retail chicken meat [16],[17]. NDM-producing Enterobacteriaceae isolated from human clinical setting were recently reported in Egypt, Morocco, Oman, United Arab Emirates, and Iran [19,40–42].

The predominant CTX-M sequence type was CTX-M-15, amounting to nearly 90%, while CTX-M-14 accounted for less than 5% of the CTX-M producing isolates. A study on Dutch retail chicken meat revealed that CTX-M-15 was not detected and CTX-M-1 was the most prevalent CTX-M ESBL type [9]. Another study of broiler chickens in Great Britain found that CTX-M-1 was the most common CTX-M sequence type followed by CTX-M-15 [38].

Phylogenetic analysis of *E*. *coli* isolates revealed that the vast majority (80%) of the ESBL-EC belonged to phylogroups A and B1, which include *E*. *coli* isolates of low virulence and commensal origin. This finding elucidates the pivotal silent role played by these commensal isolates in the spread of ESBL resistance genes. On the other hand, non-ESBL-producing isolates belonged mainly to the commensal phylogroup B1 and, to lesser extents, to phylogroups B2 and A, while the minority of isolates were phylogroup D.

In conclusion, this is the first study from Egypt showing high rates of ESBLs and carbapenemases (65.09% and 11.32%, respectively) in Enterobacteriaceae isolated from retail chicken meat. These results raise serious concerns about public health and food safety as retail meat could serve as a reservoir for these resistant bacteria which could be potentially transferred to humans through the food chain.
